# Apixaban for the treatment of saphenous vein graft thrombosis presenting as unstable angina: a case report

**DOI:** 10.1186/s12959-017-0133-5

**Published:** 2017-04-04

**Authors:** Makoto Saigan, Tsuyoshi Isawa, Tatsushi Ootomo

**Affiliations:** Department of Cardiology, Sendai Kousei Hospital, 4-15 Hirose-machi, Sendai, 980-0873 Japan

**Keywords:** Apixaban, Saphenous vein graft thrombosis, Unstable angina

## Abstract

**Background:**

Saphenous vein graft thrombosis can present as unstable angina. However, percutaneous coronary intervention for saphenous vein graft lesions poses a high risk of slow flow related to the procedure. Here we present the utilization of the novel oral anticoagulant, apixaban, in the treatment of unstable angina with extensive saphenous vein graft thrombus, leading to considerable thrombus resolution and eliminating the need of percutaneous coronary intervention.

**Case presentation:**

A 72-year-old man with 3-vessel coronary artery bypass graft surgery using a saphenous vein graft and a left internal mammary artery, performed 25 years earlier, presented at our hospital with recurrent chest tightness. The echocardiography showed regional hypokinesis of the post-lateral wall with moderate left ventricular dysfunction, which had not been previously confirmed. Coronary angiography showed obstruction of the saphenous vein graft with a large thrombus burden. The left internal mammary artery was patent and other natives were the same as they had been 3 years ago. He was diagnosed with unstable angina due to acute saphenous vein graft thrombosis. Instead of percutaneous coronary intervention, he was treated with apixaban 5 mg twice a day. The angiography 3 weeks after starting apixaban showed considerable resolution of the thrombus and opening of the saphenous vein graft.

**Conclusions:**

Apixaban could become a viable treatment option for acute saphenous vein graft thrombosis.

**Electronic supplementary material:**

The online version of this article (doi:10.1186/s12959-017-0133-5) contains supplementary material, which is available to authorized users.

## Background

Saphenous vein graft (SVG) occlusion with thrombus formation is a substantial cause of acute coronary syndrome, including unstable angina, in patients undergoing coronary artery bypass graft (CABG) surgery [1]. Percutaneous coronary intervention (PCI) is considered to be a first-line option [2]. However, SVG angioplasty is associated with frequent periprocedural complications due to distal embolization [3–5]. Therefore, the therapeutic approach in unstable angina due to SVG thrombosis is still controversial. Here we describe a case demonstrating that apixaban, a novel oral anticoagulant, was effective for unstable angina due to SVG thrombosis and might be an alternative to PCI.

## Case presentation

**Fig. 1:**
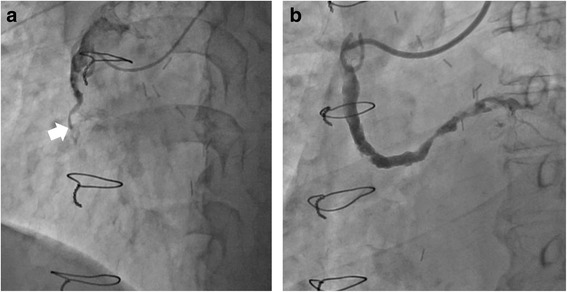
Coronary angiography before (**a**) and after apixaban (**b**). **a** A saphenous venous graft (SVG) to right coronary artery was almost occluded with a large thrombosis burden (*white arrow*). **b** After 3 weeks with apixaban treatment, the SVG was opened


A hypertensive and diabetic 72-year-old man with 3-vessel CABG surgery, SVG to the first diagonal branch of the left anterior descending artery (LAD) and to the obtuse marginal branch of the left circumflex artery (sequential grafting) and the left internal mammary artery (LIMA) to the distal LAD, performed 25 years earlier, presented at our hospital complaining of recurrent chest tightness over the past week. On physical examination, there were no remarkable findings. An electrocardiography showed a normal sinus rhythm at a rate of 78/min with a complete right bundle branch block, but no significant ST-T change was found compared with the findings of his previous electrocardiography. Regional hypokinesis of the post-lateral wall with moderate left ventricular dysfunction, which had not been previously confirmed, was also observed. Cardiac enzymes were not elevated. Coronary angiography showed the obstruction of SVG with a large thrombus burden (Fig. 1a, Movie 1). Although LAD was chronically occluded from the ostium, there was no significant change compared with his condition 3 years ago. LIMA to LAD was patent, and other natives were the same as they had been 3 years ago. He was diagnosed with unstable angina due to acute SVG thrombosis. Instead of PCI, he was treated with apixaban 5 mg twice a day for 3 weeks in addition to aspirin 100 mg once a day, which was already being taken. We added only apixaban and not a P2Y12 inhibitor considering the high risk of bleeding and the mechanism of thrombotic occlusion. After coronary angiography had been performed and PCI was selected, we planned to load a P2Y12 inhibitor. The angiography 3 weeks after starting apixaban showed a considerable resolution of the thrombus and an opening SVG (Figure 1b, Movie 2). The coronary flow of the final angiography almost reached TIMI grade 3 flow, and there was no significant stenosis in SVG. Therefore, we did not move on to PCI due to the fear of distal embolization. The rest of the patient’s stay in the hospital was uneventful, and he was discharged 1 week after the procedure.

A saphenous venous graft (SVG) to right coronary artery was almost occluded with a large thrombosis burden. (MOV 7060 kb)
After 3 weeks with apixaban treatment, the SVG was opened. (MOV 4498 kb)


## Discussion

We found 2 important clinical issues. First, apixaban can quickly remove thrombosis of SVG and can help avoid PCI. Second, apixaban safely operates with a relatively low risk of bleeding complications compared with warfarin.

SVG thrombosis is called the “deep vein thrombosis” of the heart and its possible pathophysiology is assumed to be the same as that of deep vein thrombosis [6]; therefore, novel oral anticoagulants (NOACs) were effective for SVG thrombosis, as shown in the present case. In addition, the resolution of the thrombus with apixaban was rapid (within just 3 weeks). Therefore, apixaban could become a viable option for acute SVG thrombosis presenting as unstable angina, where PCI has risk for distal embolization and rapid thrombus resolution is required.

The second important point is that apixaban is relatively safe with a low risk of bleeding complications. Compared with warfarin, which failed to show high SVG primary patency rates [7] and increased major bleeding [8], apixaban has lower rates of major bleeding [9, 10]. It should be mentioned that among NOACs, apixaban alone reveals not only significantly reduced major bleeding but also greater net clinical benefit in patients with venous thromboembolism [11]. Therefore, it is remarkable that apixaban was also effective in patients with acute SVG thrombosis, as shown in the present case, although we should not ignore the fact that apixaban itself is associated with a potential risk of bleeding.

## Conclusions

We used apixaban instead of PCI for the treatment of acute SVG thrombosis presenting as unstable angina. This novel approach, which is likely safer than warfarin and may be safer and more effective than mechanical approaches, deserves further study as a treatment for acute SVG thrombosis.

## Abbreviations

CABG: Coronary artery bypass graft
LAD: Left anterior descending artery
LIMA: Left internal mammary artery
NOACs: Novel oral anticoagulants
PCI: Percutaneous coronary intervention
SVG: Saphenous vein graft

## References

[1] Chen L, Théroux P, Lespérance J, Shabani F, Thibault B, De Guise P (1996). Angiographic features of vein grafts versus ungrafted coronary arteries in patients with unstable angina and previous bypass surgery. J Am Coll Cardiol.

[2] Lee MS, Park SJ, Kandzari DE, Kirtane AJ, Fearon WF, Brilakis ES (2011). Saphenous vein graft intervention. JACC Cardiovasc Interv.

[3] Holmes DR, Topol EJ, Califf RM, Berdan LG, Leya F, Berger PB (1995). A multicenter, randomized trial of coronary angioplasty versus directional atherectomy for patients with saphenous vein bypass graft lesions. CAVEAT-II Investigators. Circulation.

[4] Pratsos A, Fischman DL, Savage MP (2001). Restenosis in saphenous vein grafts. Curr Interv Cardiol Rep.

[5] Keeley EC, Velez CA, O’Neill WW, Safian RD (2001). Long-term clinical outcome and predictors of major adverse cardiac events after percutaneous interventions on saphenous vein grafts. J Am Coll Cardiol.

[6] Pothineni NV, Bahia A, Gobal F, Ahmed Z, Uretsky BF, Hakeem A (2015). DVT“ of the Heart: A ”Novel. Treatment for an Old Problem. JACC Cardiovasc Interv.

[7] Post Coronary Artery Bypass Graft Trial Investigators (1997). The effect of aggressive lowering of low-density lipoprotein cholesterol levels and low-dose anticoagulation on obstructive changes in saphenous-vein coronary-artery bypass grafts. N Engl J Med.

[8] McEnany MT, Salzman EW, Mundth ED, DeSanctis RW, Harthorne JW, Weintraub RM (1982). The effect of antithrombotic therapy on patency rates of saphenous vein coronary artery bypass grafts. J Thorac Cardiovasc Surg.

[9] Agnelli G, Buller HR, Cohen A, Curto M, Gallus AS, Johnson M (2013). Oral apixaban for the treatment of acute venous thromboembolism. N Engl J Med.

[10] Granger CB, Alexander JH, McMurray JJ, Lopes RD, Hylek EM, Hanna M (2011). Apixaban versus warfarin in patients with atrial fibrillation. N Engl J Med.

[11] Kakkos SK, Kirkilesis GI, Tsolakis IA (2014). Editor’s Choice - efficacy and safety of the new oral anticoagulants dabigatran, rivaroxaban, apixaban, and edoxaban in the treatment and secondary prevention of venous thromboembolism: a systematic review and meta-analysis of phase III trials. Eur J Vasc Endovasc Surg.

